# Clinical Impact of Blood Glucose Monitoring Accuracy: An In-Silico Study

**DOI:** 10.1177/1932296817710474

**Published:** 2017-06-01

**Authors:** Enrique Campos-Náñez, Kurt Fortwaengler, Marc D. Breton

**Affiliations:** 1 Center for Diabetes Technology, University of Virginia, Charlottesville, VA, USA; 2 Roche Diabetes Care, Mannheim, Germany

**Keywords:** accuracy, blood glucose meters, clinical outcomes

## Abstract

**Background:**

Patients with diabetes rely on blood glucose (BG) monitoring devices to manage their condition. As some self-monitoring devices are becoming more and more accurate, it becomes critical to understand the relationship between system accuracy and clinical outcomes, and the potential benefits of analytical accuracy.

**Methods:**

We conducted a 30-day in-silico study in type 1 diabetes mellitus (T1DM) patients using continuous subcutaneous insulin infusion (CSII) therapy and a variety of BG meters, using the FDA-approved University of Virginia (UVA)/Padova Type 1 Simulator. We used simulated meter models derived from the published characteristics of 43 commercial meters. By controlling random events in each parallel run, we isolated the differences in clinical performance that are directly associated with the meter characteristics.

**Results:**

A meter’s systematic bias has a significant and inverse effect on HbA1c (*P* < .01), while also affecting the number of severe hypoglycemia events. On the other hand, error, defined as the fraction of measurements beyond 5% of the true value, is a predictor of severe hypoglycemia events (*P* < .01), but in the absence of bias has a nonsignificant effect on average glycemia (HbA1c). Both bias and error have significant effects on total daily insulin (TDI) and the number of necessary glucose measurements per day (*P* < .01). Furthermore, these relationships can be accurately modeled using linear regression on meter bias and error.

**Conclusions:**

Two components of meter accuracy, bias and error, clearly affect clinical outcomes. While error has little effect on HbA1c, it tends to increase episodes of severe hypoglycemia. Meter bias has significant effects on all considered metrics: a positive systemic bias will reduce HbA1c, but increase the number of severe hypoglycemia attacks, TDI use, and number of fingersticks per day.

Glycemic control in patients with type 1 and type 2 diabetes is a life-long effort driven by information. Self-monitoring of blood glucose (SMBG) systems are fundamental in optimizing glycemic control for these patients and as such, blood glucose (BG) meter technology has experienced significant progress and is part of most clinical guidelines for glycemic control. While the system accuracy of BG meters has been widely studied,^1^ little is known about the impact of accuracy on clinical outcomes. The maturity of mathematical models representing glucose metabolism in health and diabetes^2^ and the increasing acceptance of computer simulation to predict the impact of therapy modification in type 1 diabetes mellitus (T1DM)^3,4^ have led to regulatory acceptance of modeling and simulation, “for approximation of human glucose/insulin utilization, interstitial sensor performance, and subcutaneous insulin delivery,” which included replacement for animal trials in the preclinical testing of artificial pancreas systems.^3^ The University of Virginia (UVA)/Padova simulation platform is now commonly used for testing medical device performance as well as novel treatment strategies in T1DM;^5-12^ the platform is frequently augmented by new physiological processes.^13-17^

In this study, we aim to quantify the clinical impact of BG monitoring system accuracy using the latest UVA/Padova simulation platform by (1) constructing models that replicate the characteristics of commercially available BG monitoring systems, (2) modeling type 1 subject behavior related to meals and self-treatment, and (3) constructing a simulation to track T1DM patients over 30 days. Each simulated patient uses each of the available BG monitoring systems in turn, and simulation results are used to estimate clinical outcomes such as HbA1c, severe hypoglycemia, and other clinically relevant parameters. Finally, we create regression models that relate the characteristics of BG monitoring systems to each of the clinical outcomes.

## Materials and Methods

**Figure 1.**
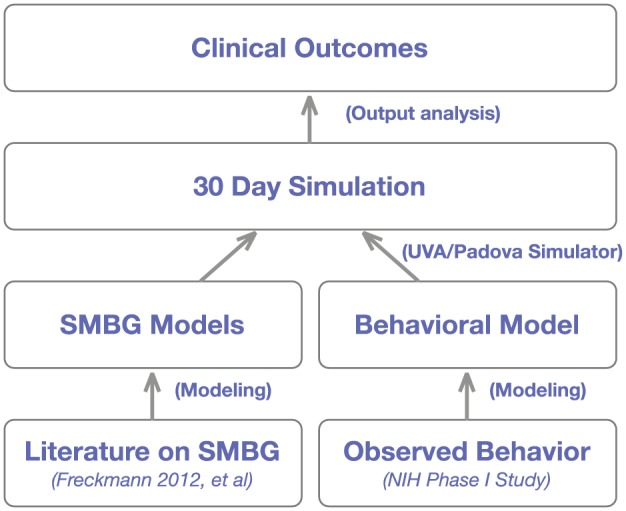
Project approach to estimating clinical impacts of meter accuracy.

Building on the work relating accuracy to clinical outcomes,^18-30^ we assess the effect of SMBG systems on clinical outcomes. An overview of the process followed in this work is described in Figure 1. (1) Our approach relied on existing literature and publicly available data to model commercially available BG meters as well as self-treatment behavior observed in continuous subcutaneous insulin infusion (CSII) therapy; (2) the resulting models, described in the following sections were incorporated in the UVA/Padova Type 1 Diabetes Simulator,^13,31^ and used to create a thirty day long simulation where each in-silico subject in the population used in turn each of the available commercial meters; and (3) quality of glucose control observed by each of the subject/meter combination was estimated using standard metrics such as HbA1c, severe hypoglycemia, number of fingersticks used, and an estimate of total daily insulin (TDI). The following sections describe in detail each of the steps.

### Modeling BG Monitoring Systems

A comprehensive study^1^ reported the system accuracy of 43 commercial BG monitoring systems. Accuracy was defined in terms of percentages of measurements within certain tolerances of the reference measurement, split between low and high glucose ranges (<100 mg/dl, ≥100 mg/dl, respectively). The BG monitoring systems are modeled using a Johnson transform of a standard normal distribution where parameters are selected to optimally match the reported measurement percentage errors reported in literature. Specifically, in the low glucose range (<100 mg/dl), the parameters of a Johnson distribution

$$Y_{L}=\chi_{L}+\lambda_{L}\sinh\left(\frac{N-\gamma_{L}}{\delta_{L}}\right),$$

are estimated so that $P\left(Y_{L}\leq l_{i}\right)\approx pl_{i}$, where $l_{i}$ is one of the threshold in 15, 10, 5 mg/dl, and $pl_{i}$ is the reported percentage of measurements within that threshold.^1^ Here *N* is a standard normal variate. A similar approach is used to fit parameters $\chi_{H},\lambda_{H},\gamma_{H},\delta_{H}$ to generate a simulated measurement *N* for the corresponding high glucose range (≥100 mg/dl), noting that in that case thresholds are expressed in percentages, that is, 15%, 10%, and 5% from the reference measurement, and we seek to match $P\left(\frac{Y_{H}}{100}\leq h_{i}\right)\approx ph_{i}$. It is important to note that given a set percentage $pl_{i}$ there are two symmetric distributions satisfying these properties. The sign of the distribution is resolved by matching systematic biases reported^1^ for each BG monitoring system. The complete set of parameters required to represent a specific meter is $\left(\chi_{L},\lambda_{L},\gamma_{L},\delta_{L},\chi_{H},\lambda_{H},\gamma_{H},\delta_{H}\right)$.

#### Use in Simulation

**Figure 2.**
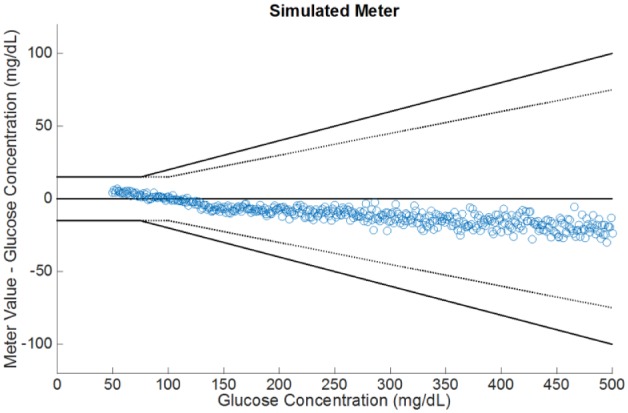
Example simulated glucose readings using our modeling approach.

Simulated measurements given a reference glucose value G can be obtained from a standard normal variate *N* by computing measurement as

$$SMBG=\left(1-\alpha \right)\left(G+Y_{L}\right)+\alpha G\left(1+\frac{Y_{H}}{100}\right),$$

where $\alpha =max\left\{0,min\left\{1,\frac{G-70}{130-70}\right\}\right\}.$ The above expression returns the low glucose measurement $G+Y_{L}$ whenever the reference G<70, it will return the high glucose measurement $G\left(1+\frac{Y_{H}}{100}\right)$ when G≥130, and a mix of the two in the range 70≤G<130. In Figure 2, we show the result of such an approach.

### Modeling Behavior

**Figure 3.**
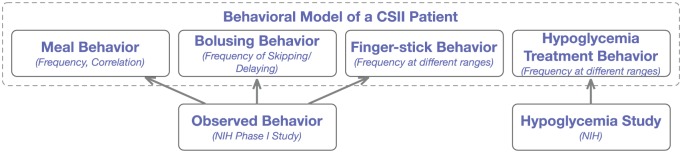
Description of the elements of the basic behavioral model.

The behavioral model adopted in this work consisted of the components described in Figure 3, namely (1) a meal behavior component that describes eating amount, times, and correlations; (2) a bolusing behavior component that describes the conditions under which a bolus is self-administered; (3) a fingerstick behavior component describing how frequently subjects fingerstick and under what conditions; and (4) a hypoglycemia self-treatment behavior. Before describing in detail each of these components, we briefly describe the data sources used to design and parameterize our behavioral model.

#### Data Sources

We used archived deidentified data collected during the project funded by National Institutes of Health/National Institute of Diabetes and Digestive and Kidney Diseases grant RO1 DK 085623 (see clinicaltrial.gov for clinical trial registration number NCT01434030). Sixty insulin pump users with type 1 diabetes were recruited and asked to wear a CGM device for a month, simultaneously recording SMBG, CGM, and insulin pump data, as well as information about meals and physical activity. Fifty-six participants completed the data collection and contributed to the database. The demographic characteristics of these subjects were as follows: 21-65 years of age, with a mean (SD) age of 41 (12.2) years; duration of type 1 diabetes of at least 2 years, with a mean duration of 24.1 (11.0) years; use of an insulin pump for at least 6 months, with a mean interval of 10 (5.8) years; and active use of a bolus calculator function. Mean (SD) hemoglobin A1c level was 7.7% (1.2%), 59% were female, the majority (95%) were white, and 50% were employed in professional occupations. The database building protocol was approved by the local Institutional Review Board; study details were previously published.^32^ Data collection was designed to interfere minimally and reduce as much as possible burden, distractions or alterations to participants’ typical daily routines and allow them to maintain typical daily behaviors.

#### Meal Behavior

**Figure 4.**
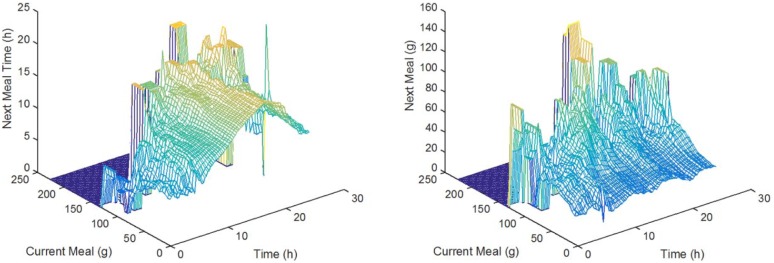
Relationship between consecutive meal times and sizes, using values of $\delta_{t}=15\left(min\right),and\;\delta_{a}=20$(g).

To account for interrelationships between the timing and amount of consecutive meals that is observed in real life, we adopt bootstrap sampling in the following sense: consider a database of meals where for meal m we have the associated meal time of day and amount $t_{m},a_{m}$, respectively. We assume meal m+1 follows meal *m* (ie, meals are ordered by time). Using this information, we can construct the set of meals

$$M\left(t,a\right)=\left\{m|t_{m}\in \left(t-\delta_{t},t+\delta_{t}\right),a_{m}\in \left(a-\delta_{m},a+\delta_{m}\right)\right\},$$

that is, meals that took place at approximately time t of a size approximately equal to a. As illustrated in Figure 4, both the size and the timing of a meal depend on the timing and size of the meal just prior. To mimic this behavior, given a meal time *N* and size a, meals are uniformly sampled from the set $M^{+}\left(t,a\right)$, the set of meals following meals in set M(t,a), where $M^{+}\left(t,a\right)=\left\{m|m-1\in M\left(t,a\right)\right\}$. Based on our data sets, the time between meals has a mean of 3.64 hours (standard deviation of 2.73), median of 3.16 hours, where the lowest 5% of these times fall below 25 minutes, and the 95% fall under 8.75 hours. The average meal size is 39.6 g (24.8 g).

#### Bolus and Fingerstick Behavior

**Figure 5.**
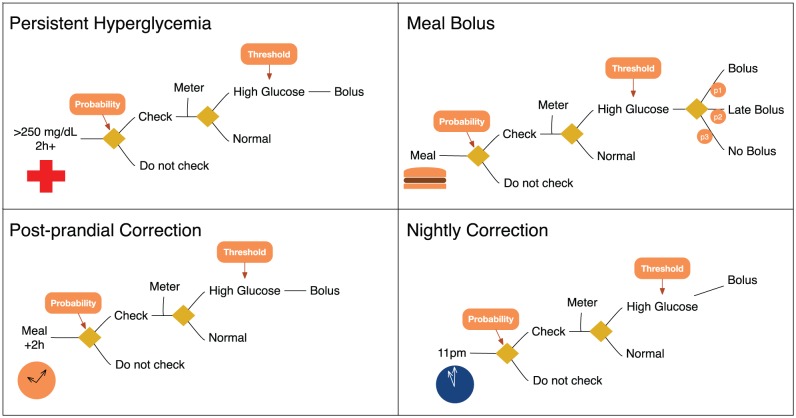
Behavioral model of bolus and fingerstick behavior.

Fingerstick and bolus behavior is modeled according to Figure 5. The subject responds probabilistically to a triggering event (eg, meal or hyperglycemia), and applies a bolus if necessary according to functional therapy parameters. For example, in the top-left of Figure 5, we illustrate how the in-silico subject responds to persistent hyperglycemia (two consecutive hours of high glucose >220 mg/dl): a fingerstick is applied with a fixed probability, followed by the appropriate correction. A slightly more detailed model was used to model meal bolus behavior, where patients may (randomly) decide to ignore or delay a meal bolus.

#### Hypoglycemia Treatment

Hypoglycemia is treated according to the following rule. This response approximates behavioral data from the data sets reported above. As observed, the size of hypo treatments will increase as treatments are repeated. Moreover, a lower glycose level will result in an increase in rescue size. More specifically, a low glucose concentration level (<80 mg/dl) will trigger a fingerstick with probability $e^{\left(35-BG\right)/8}$ every half-hour.^12^ This implies that when BG lowers to 35 mg/dl, it will be detected for sure, but on the other hand, if BG = 70, the probability of triggering an SMBG check within 15 minutes is approximately 20%. If low glucose is indeed detected, rescue carbs are administered according to hypoglycemia level and number of rescues applied recently (within 30 min), as described in Table 1. For example, a subject weighing 90 kg with a glucose reading of 55 mg/dl that has received 3 consecutive rescues (within 30 min), will receive an additional one of 0.5 × 90 = 45 g. On the other hand, suppose that the first rescue the subject received was given when her glucose was 65 mg/dl, will receive 0.2 × 90 = 18 g carbohydrates.

**Table 1.** Rescue Carb Schedule in Grams/kg.

	Number of consecutive rescues		
Hypoglycemia level	1	2	3+
<70 mg/dl, ≥60 mg/dl	0.2	0.4	0.6
<60 mg/dl, ≥50 mg/dl	0.4	0.8	0.8
<50 mg/dl	0.6	1.2	1.2

### In-Silico Evaluation

A 30-day scenario was designed based on behavioral models described in the previous sections, and used to generate the glycemic response for each 100 in-silico subject of the T1D adult population in the UVA/Padova simulator, using each BG monitoring system modeled in our database, a total of 43 meters, together with an ideal meter (neither bias nor error). Given the natural variability of the process and to improve estimates of our metrics, 30 replicates of each combination of subject-BG monitoring system were simulated, resulting in more than 10 000 simulated patient years. Random seeds were controlled across subjects and meters to minimize variance of the estimates and accurately isolate the effect of meter accuracy on glycemic control. In other words, each subject will experience the same meal sequence, bolus decisions, and other behavioral factors. Thus, differences across scenarios are only due to meter characteristics.

#### Clinical Outcome Estimates

For each simulation, we computed an estimated HbA1c (using a commonly used linear regression model relating average glucose to HbA1c^33^) and severe hypoglycemia events (via a model relating LBGI and severe hypoglycemia events; see Table 2 in Kovatchev et al^34^). We note that severe hypoglycemia, defined as a glucose level such that the patient will require assistance, is not explicitly simulated, but later estimated. In addition, TDI and daily fingerstick use were computed directly from the simulation output.

**Table 2.** Metric Estimates Obtained by Averaging Simulation Outputs Across Subjects.

Meter/metric	HbA1c (%)	Severe hypoglycemia (events/6 months)	TDI (U)	Fingerstick count (meas./day)	Bias (mg/dl)	Error (fraction ARD > 5%)
Meter 1	8.9	1.1	41.3	8.0	0.3	0.2
Meter 2	8.7	1.4	41.5	8.4	0.0	0.4
Meter 3	8.9	1.1	41.2	8.0	−3.7	0.4
Meter 4	8.7	1.4	42.3	8.4	−6.8	0.3
Meter 5	8.7	1.4	43.2	8.4	4.4	0.2
Meter 6	8.7	1.4	42.5	8.3	−3.0	0.3
Meter 7	8.9	1.2	41.6	8.1	−7.3	0.3
Meter 8	8.6	1.6	43.6	8.6	5.6	0.3
Meter 9	8.6	1.6	42.8	8.5	2.2	0.3
Meter 10	8.9	1.1	42.1	8.1	−3.6	0.4
Meter 11	8.5	2.0	43.8	9.0	7.1	0.7
Meter 12	8.6	1.7	43.3	8.7	4.1	0.4
Meter 13	9.0	1.0	41.5	7.9	−10.3	0.6
Meter 14	8.7	1.5	42.8	8.4	0.0	0.4
Meter 15	8.9	1.2	41.0	8.1	4.9	0.5
Meter 16	9.0	1.1	42.9	8.0	−10.9	0.7
Meter 17	8.7	1.4	41.3	8.4	3.2	0.1
Meter 18	8.9	1.1	41.2	8.1	0.8	0.1
Meter 19	8.4	2.3	47.3	9.3	15.1	0.7
Meter 20	9.0	1.1	41.2	8.0	−3.6	0.6
Meter 21	8.7	1.6	42.0	8.5	0.0	0.6
Meter 22	9.0	1.0	42.2	8.0	−3.4	0.6
Meter 23	8.5	1.8	44.5	8.8	8.9	0.6
Meter 24	8.5	2.2	43.9	9.3	21.0	0.7
Meter 25	9.2	1.0	42.0	7.9	−21.9	0.8
Meter 26	8.4	2.1	43.3	9.2	16.8	0.7
Meter 27	8.5	1.9	44.5	9.0	12.2	0.6
Meter 28	8.5	2.0	45.1	9.0	11.0	0.6
Meter 29	8.5	1.9	43.8	8.9	0.7	0.6
Meter 30	8.9	1.1	40.5	8.1	6.4	0.5
Meter 31	9.0	1.1	42.1	8.0	−9.0	0.6
Meter 32	9.0	1.0	41.3	7.9	−10.0	0.6
Meter 33	9.0	1.1	41.0	8.0	−9.5	0.6
Meter 34	8.6	1.8	44.4	8.8	3.9	0.5
Meter 35	8.6	1.7	43.2	8.7	9.5	0.4
Meter 36	8.5	2.0	43.4	9.0	15.2	0.6
Meter 37	8.9	1.1	40.8	8.0	−4.8	0.5
Meter 38	9.1	0.9	41.3	7.9	−12.3	0.7
Meter 39	8.4	2.3	46.8	9.4	23.6	0.7
Meter 40	8.7	1.5	42.1	8.4	9.4	0.4
Meter 41	8.7	1.5	42.8	8.4	−3.0	0.5
Meter 42	8.5	1.8	43.2	8.8	10.8	0.5
Meter 43	8.7	1.6	42.4	8.6	12.0	0.5
**Ideal (error free)**	**8.8**	**1.4**	**41.8**	**8.4**	**0.0**	**0.0**

For each combination of subject s, meter m, and replicate r, we have an estimate for $HbA1c_{s,m}^{r}$, an estimate $SH_{s,m}^{r}$ of severe hypoglycemia events per person per 6 months, an estimate $TDI_{s,m}^{r}$ of average TDI used, as well as $FS_{s,m}^{r}$ a fingerstick count. In addition, we define accuracy of a meter in terms of two following characteristics used to describe our results.

##### Error

Fraction of measurements whose absolute relative difference is >5%. Formally, if we have a collection of n measurements ${{SMBG}^{m}}_{i}$ obtained with meter m, paired with reference values $BG_{i}$, with i=1,2,…,n, we define

$$Error_{m}=\frac{1}{n}\overset{n}{\underset{i=1}{\sum }}1_{\left\{\left|\frac{{{SMBG}^{m}}_{i}-BG_{i}}{BG_{i}}\right|>.05\right\}},$$

where $1_{A}$ is the indicator of event A.

##### Bias

The average difference between measurement and reference, that is,

$$Bias_{m}=\frac{1}{n}\overset{n}{\underset{i=1}{\sum }}({{SMBG}^{m}}_{i}-BG_{i}).$$

In Table 2, we report population means for each of these metrics and meters. For example, given a fixed meter m, we denote the average HbA1c across all subjects and replicates as

$${\bar{HbA1c}}_{m}=E\left[HbA1c_{S,m}^{R}\right]\equiv \frac{1}{100\;\times 30}\overset{100}{\underset{s=1}{\sum }}\overset{30}{\underset{r=1}{\sum }}HbA1c_{s,m}^{r}.$$

Correspondingly, we denote by ${\bar{SH}}_{m}$, ${\bar{FS}}_{m}$, and ${\bar{TDI}}_{m}$, the average across all subjects and replicates of severe hypoglycemia episodes, fingerstick count, and TDI, respectively. Similarly, we denote per subject averages (across replicates) as ${\bar{HbA1c}}_{s,m}$, ${\bar{SH}}_{s,m}$, ${\bar{FS}}_{s,m}$, and ${\bar{TDI}}_{s,m}$.

## Results

In Table 2 we report all simulation results in terms of HbA1c, severe hypoglycemia, TDI, and fingerstick count. Entries in Table 2 represent the average value of each metric across 30 replicates of 100 subjects in the adult population all using each of the 43 glucose meter models. The table also reports the average bias and errors obtained for the corresponding meter, as observed in the simulation.

### Response of HbA1c to Error and Systematic Bias

**Figure 6.**
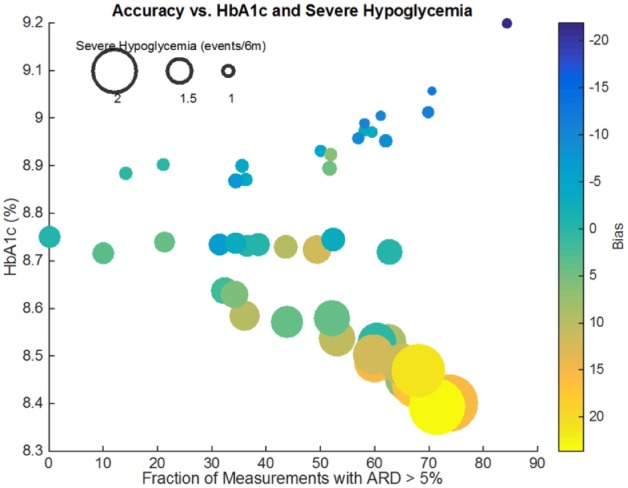
Relationship between error, bias, HbA1c, and severe hypoglycemia. Each meter is represented by a colored dot. The *x*-coordinate represents error, while the *y*-coordinate represents the resulting HbA1c. The size of the dot is proportional to the number of severe hypoglycemia events in 6 months, while the dot’s color shows the meter’s systematic bias.

Meter error has a marginal effect on HbA1c. In fact, as we will show in our next section, a linear regression model shows that the coefficient corresponding to meter error is not significant. On the other hand, the data shows a clear inverse relationship between meter systematic bias and HbA1c (as illustrated in Figure 6). Meters with positive bias (represented as orange-yellow dots), will tend to reduce HbA1c while at the same time increasing the expected number of severe hypoglycemia events.

### Response of Severe Hypoglycemia to Error and Systematic Bias

The effect of error, which was negligible on HbA1c, is significant on severe hypoglycemia: higher error rates are associated with a higher number of severe hypoglycemia. These relationships will be further formalized in the following sections where we show that a simple linear regression model can explain most of the results shown here.

### Other Relationships

TDI, as well as fingerstick count, shows similar relationships to severe hypoglycemia, that is, from a reference of 41.8U of TDI for an ideal meter, high error rates as well as positive bias will result in higher daily insulin use. For example, meter 19 with a positive bias of 15.1 mg/dl and an error of 0.7 (on a scale of 0 to 1), increases insulin use to 47.3U per day. A similar relationship is observed for fingerstick counts.

### A Model Relating Accuracy and Clinical Outcomes

We propose a linear model

$$\begin{array}{l} {\bar{HbA1c}}_{s,m}\sim a_{0}+a_{1}{\bar{error}}_{m}+a_{2}{\bar{bias}}_{m}+a_{3}{\bar{HbA1c}}_{s,0}+\\ \;a_{4}{\bar{FSCount}}_{m}+a_{5}{\bar{error}}_{m}{\bar{bias}}_{m}, \end{array}$$

where ${\bar{HbA1c}}_{s,0}$ is the average HbA1c observed by subject *N* when using the ideal (error-free) meter, and ${\bar{FSCount}}_{m}$ is average number of fingersticks used by subject using meter m. Similar models were created for TDI, and for daily fingerstick count (excluding in this case ${\bar{FSCount}}_{m}$). Results are reported in Table 3. Severe hypoglycemia was modeled using a nonlinear (logistic) model. First, we define the linear model

$$\begin{array}{l} SHL_{m}\sim a_{0}+a_{1}{\bar{error}}_{m}+a_{2}{\bar{bias}}_{m}+\\ \;a_{4}{\bar{SH}}_{0}+a_{3}{\bar{error}}_{m}{\bar{bias}}_{m}, \end{array}$$

**Table 3.** Regression Model Parameters Relating Error, Bias, Fingerstick Counts, and per Subject Baseline to Clinical Outcomes.

Coefficient\value	HbA1c***	Severe hypoglycemia***	TDI***	Fingerstick count***
Intercept (a_0_)	0.945***	−1.325***	3.0267***	−0.134***
Error (a_1_)	0.00882	0.314***	2.573***	0.492***
Bias (a_2_)	−0.00681**	0.017***	0.0347	0.0263***
Baseline (a_3_)	0.91***	0.747***	0.824***	0.988***
Fingersticks (a_4_)	−0.0155***	0.182***	0.454***	n/a
Error x Bias (a_5_)	−0.0165*	0.0166**	0.0747	0.0192*
Adjusted R-squared	0.77	0.953	0.771	0.949
θ_1_	N/A	9.0668***	N/A	N/A
θ_2_	N/A	4.497***	N/A	N/A
θ_3_	N/A	1.535***	N/A	N/A

* *P* < .05. **P < .01. ***P < .001.

and the prediction for severe hypoglycemia takes the form

$$SH_{m}\sim \frac{\theta_{1}}{1+e^{\left(\theta_{2}-SML_{m}\right)/\theta_{3}}}.$$

and the prediction for severe hypoglycemia takes the form

## Discussion

Based on our simulations, we could show a clear relationship between the system accuracy of a BG monitoring system and the resulting quality of glucose control. More specifically, a meter with a large error rate will tend to increase glucose variability and therefore episodes of severe hypoglycemia. The BG monitoring systems with large systematic bias, on the other hand, will have a dual and symmetric effect: the effect on HbA1c will be inversely proportional while incidence of severe hypoglycemia will be proportional to systematic bias. A previous retrospective study has similar conclusions,^35^ showing for instance that meters with readings consistently higher than reference can significantly increase hypoglycemic coma episodes.

Generalization of these results is somehow limited by the use of models mimicking patient’s behavior during a relatively small scale (n = 55, 1 month) clinical study; while the protocol was designed to minimize contact with participants and perturbations of their treatment behaviors, this group may still have exhibited treatment behaviors that were more compliant than ones observed for the population at large. Measurements frequency in particular may impact our results, particularly the balance between bias and variance of the error. It is important to note that while the observed BGM frequency was high it was equivalent in all simulation, leading to changes in hypoglycemia and HbA1c that were purely driven by BGM error characteristics.

In summary, our results demonstrate that BG monitoring systems compliant with most conditions of the ISO 15197 (2013) standard have only limited impact on HbA1c, SHE, insulin utilization and SMBG frequency, whereas systems not meeting the standards can have significant clinical influence on one or several of these outcomes. For the HbA1c values, the increases could reach approximately 0.4% while the number of annual SHE could increase by up to 1.7 cases per year. In addition, the insulin consumption could increase by up to 5.5 units/day and the number of fingersticks by up to 1.0 tests/day.

Importantly, these are not limited to just one specific SMBG system; rather, they apply to several systems not meeting the most recent version of ISO standard 15197. These findings not only offer important guidance to both clinicians and individuals with diabetes when selecting an appropriate SMBG system but they provide a basis for estimation of the economic impact of SMBG system inaccuracy, which will be presented in a subsequent publication. The relationship between CGM-MARD and the ISO 15197 (2013) standard in nonadjunctive use is also critical. As recent studies have shown, it is difficult to directly relate MARD levels to the ISO 15197:2013 standard. A recent study^36^ showed that MARDs of 3.25% and 5.25% are required to achieve ISO standard with probability one.

## Conclusion

In this study, we present a new approach to estimate the impact of BG monitoring system accuracy on clinical outcomes. This approach allows us to leverage recent advances in the simulation of the glucose-insulin metabolism as well as new behavioral models of type 1 diabetes patients to assess the clinical impact of inaccurate glucose meters in everyday use. Patients under CSII therapy will receive benefits from increases in accuracy in both CGM and BGM technologies. These effects will be observed independent of the mode of BGM use, for example, insulin-dosing or calibration only.

Although this in-silico study simulated a CSII based population, we can assume that, at least qualitatively, these results can be extended to patients using functional insulin therapy and multiple daily injections (MDI); it is still unclear how much of these results are applicable to older forms of therapy, for example, sliding scale. Our simulation study does not consider temporary basal rate adjustments, so as long as the considered basal insulins display long (~24-hour) time constants (eg, glargine), results are not likely to change. Behavioral models were trained on a combination of MDI and CSII patients, and are representative of this mixture. Further simulation studies would allow to quantify the sensitivity of our results to these behavioral parameters, in particular the response to changes in the number of treatment decisions.

An important limitation in our study is the lack of long-term behavioral adjustments. For instance, it has been observed that patients with T1DM who experience frequent episodes of hypoglycemia will adjust their therapy on their own or with assistance by their physician. Although the results presented here accurately predict short-term clinical outcomes, the long-term effects of such behavioral adaptations need to be better understood. While such behavioral adaptations are commonly observed in clinical practice, the authors are not aware of data sets with sufficient detail to properly model and simulate this behavior.
